# Enhancing the Selection of Backoff Interval Using Fuzzy Logic over Wireless Ad Hoc Networks

**DOI:** 10.1155/2015/680681

**Published:** 2015-03-23

**Authors:** Radha Ranganathan, Kathiravan Kannan

**Affiliations:** ^1^Department of Information Technology, Easwari Engineering College, Chennai 600089, India; ^2^Department of Computer Science and Engineering, Easwari Engineering College, Chennai 600089, India

## Abstract

IEEE 802.11 is the de facto standard for medium access over wireless ad hoc network. The collision avoidance mechanism (i.e., random binary exponential backoff—BEB) of IEEE 802.11 DCF (distributed coordination function) is inefficient and unfair especially under heavy load. In the literature, many algorithms have been proposed to tune the contention window (CW) size. However, these algorithms make every node select its backoff interval between [0, CW] in a random and uniform manner. This randomness is incorporated to avoid collisions among the nodes. But this random backoff interval can change the optimal order and frequency of channel access among competing nodes which results in unfairness and increased delay. In this paper, we propose an algorithm that schedules the medium access in a fair and effective manner. This algorithm enhances IEEE 802.11 DCF with additional level of contention resolution that prioritizes the contending nodes according to its queue length and waiting time. Each node computes its unique backoff interval using fuzzy logic based on the input parameters collected from contending nodes through overhearing. We evaluate our algorithm against IEEE 802.11, GDCF (gentle distributed coordination function) protocols using ns-2.35 simulator and show that our algorithm achieves good performance.

## 1. Introduction

Ad hoc network is a collection of dynamic, self-configured, and radio equipped nodes without any infrastructure. Ad hoc networks require every intermediate node to act as routers, receiving and forwarding data to every other node. This type of network is prevalently deployed in various scenarios wherein instantaneous connectivity becomes the need of the hour, either in emergency situations like a disastrous evacuation situation or in a casual get-together for presentations.

IEEE 802.11 MAC is the predominant protocol used over ad hoc networks for medium access. Binary exponential backoff algorithm (BEB) has been used by IEEE 802.11 DCF for collision avoidance. Whenever a node wants to transmit a packet, it starts sensing the medium. If the medium is idle for distributed interframe space (DIFS) period, then the node generates a backoff counter which is set for a random value between [0, CW]. After that, the backoff counter is decremented by one for every idle slot. If the channel is busy, the backoff counter is paused until the next DIFS free period. When the backoff counter reaches zero, the node starts transmission. Here the minimum and maximum values of CW are called CW_min⁡_ and CW_max⁡_ with the default values of 31 and 1023, respectively. CW is initially set to CW_min⁡_ and after every unsuccessful transmission CW is doubled with the maximum limit of CW_max⁡_. Upon a successful transmission, CW is reset to CW_min⁡_.

Bianchi [1] analyzed the saturated throughput using Markov chain model and showed that the throughput increases with the smaller number of active nodes and small CW. When the number of active nodes increases smaller CW can lead to high collision. Larger CW improves the fairness among flows but reduces the overall throughput. Random BEB algorithm of IEEE 802.11 failed to improve the fairness and throughput over heavily congested ad hoc networks. Many algorithms have been developed to tune the contention window (CW) according to the congestion status. These proposals targeted for improving either throughput or fairness or both.

The algorithms proposed in the literature can be put under two categories. They are overhearing-based and non-overhearing-based solutions. In overhearing-based solutions, each node collects information such as rate, channel utilization, and buffer status from neighbors and adapts to the new contention window (CW) according to some policy. But it is well known that contention loss occurs mainly due to hidden terminals, whereas overhearing is limited to neighbors. These methods fail to consider the status of hidden terminals. Nonoverhearing solutions enable the nodes to utilize their local information like number of idle slots, busy slots, Tx failures, and so forth, to tune its contention window (CW). However, nodes within the same transmission range assess the channel in the same way. It does not help the nodes to have differentiation and get fair channel access.

The proposed solutions realize the channel congestion status either through overhearing or local information and tunes contention window size according to some policy. After tuning, they tend to select the backoff interval randomly between [0, CW]. In one way, this random selection helps to avoid collision between the nodes that are using the same CW. But, in another way, this randomness badly changes the order and frequency of medium access among nodes due to the zero lower bound. Random selection even leads to collisions under heavy load. This scenario affects the throughput and fairness by increasing the delay and collisions. Instead of random selection, node differentiation based on their individual parameters can optimally order the medium access among competing nodes. Optimal scheduling of the medium access is a challenging task over ad hoc networks due to its distributive and dynamic nature. Every node needs to collect information about the status of contending nodes to schedule itself to access the medium. The collected information is dynamic and vague due to the ever-changing topology of the network.

In this paper IEEE 802.11 binary exponential backoff algorithm is used for selecting contention window size (CW). To the best of our knowledge, this is the first time we introduce an algorithm that computes the backoff interval within the CW limit such that it schedules the medium in a fair and efficient way without involving much overhead. It also controls the contention by ordering the nodes according to its waiting time. We make use of fuzzy logic to compute the unique backoff interval between [0, CW]. Fuzzy logic is a simple problem solving methodology that accepts vague or ambiguous input values and makes us arrive at a definite and crisp output using simple if…then…rules. These rules should reflect the exact behavior of the system.

Each node collects the input parameters queue length and waiting time from contending nodes through overhearing and stores them in a neighbor table. Every node is also responsible for advertising its input parameters (queue length—myqlen and waiting time—mywt) through request to send (RTS) message. When a node overhears this RTS message, it gains knowledge about its neighbors. This learning starts from training phase and continues. During transmission each node has to choose a count between [0, CW] and set it for the backoff counter by comparing its own input parameters with the collected information. This dynamic and vague information is applied to the membership functions to derive fuzzy variables. These fuzzy variables are fed to the fuzzy inference engine to get fuzzy output. Finally, defuzzification helps to derive at the crisp and unique backoff counter value between [0, CW].

The rest of the paper is organized as follows. In Section 2 we describe the related work and in Section 3, we brief about fuzzy logic and the steps involved and the key elements of our design in detail. We evaluate our performance against IEEE 802.11 and GDCF in Section 4 and finally we conclude our paper in Section 5.

## 2. Related Work

BEB algorithm of IEEE 802.11 [2] suffers from severe performance degradation under heavy traffic over wireless ad hoc network. It is well accepted that contention window plays vital role in improving the aggregate throughput and fairness. In this section, we review the proposals that tune the backoff interval with the goal of achieving good throughput or fairness or both. In [3], authors derived an analytical model to find optimal *P* value that reaches the theoretical throughput limit for* P*-persistent IEEE 802.11 protocol. To perform this, each node must have known the exact number of stations in the network and it depends on feedback information. To overcome this drawback, asymptotically optimal backoff (AOB) has been proposed by authors [4] to dynamically tune CW size according to the channel contention level. They probabilistically postpone transmission based on slot utilization factor. They show that their algorithm achieves theoretical capacity. In [5], authors tune the contention window based on the bit error rate of the medium. Both of the above methods need to estimate the number of active stations.

The authors of [6] use linear programming algorithm to optimize the minimum contention window size based on the channel condition (signal to noise ratio) and number of competing stations. Authors choose the access mode and CW_min⁡_ with analytical approach to optimize the throughput. They depend on network feedback to collect the channel condition status. Virtual backoff algorithm (VBA) [7] was developed using sequencing technique to reduce the number of collisions, thereby improving the throughput. However, VBA works well only in steady state where the number of nodes is fixed. VBA suffers from collisions in a dynamic scenario. In [8], authors analytically derive contention window size based on slot utilization and optimize the throughput in both saturated and nonsaturated conditions. It utilizes only local information like busy slots and free slots and does not require estimating the number of active stations. The authors of [9] propose an algorithm GDCF wherein they perform gentle decrease of contention window to reduce collision probability. They do not reset the contention window size after every successful transmission. Instead, they find optimal counter* c* and the contention window is halved after* c* consecutive successful transmission. This method reduces the collision when the number of nodes is large. Nodes need to know the number of nodes in the network to find optimal value of* c*. Authors improve both fairness and throughput using this algorithm.

In [10], authors propose a control theoretic approach to tune contention window based on the locally available information. By comparing the average number of consecutive idle slots between two transmissions against the optimal set point, this method tunes CW and achieves optimal throughput and fairness. This method uses local information, but it also depends on the number of active stations. In [11] authors achieve fairness and weighted fairness among nodes using proposed increase with synchronized multiplicative decrease that supports background transmission. In [12], channel capacity is distributed among contending nodes through overhearing. This helps in improving the fairness among nodes. MadMAC protocol in [13] achieves both fairness and throughput using limited local information like number of experienced collisions and carrier sensing information. In [14], authors use fuzzy logic to tune the contention window based on the fuzzy parameters such as busy degree of the medium and number of neighbor nodes. This approach reduces collision probability and improves throughput and also fairness. Simplified backoff algorithm (SBA) [15] uses only local information like success, collision probability to tune CW. There are only two possible sizes for CW called CW_min⁡_ (31) and CW_max⁡_ (1023). CW is assigned to CW_min⁡_ and CW_max⁡_ during light load and heavy load, respectively. Authors claim to improve fairness and throughput. But this algorithm increases the delay due to the large CW. All of the above algorithms concentrate on tuning contention window according to the congestion level of the medium and they finally select the backoff interval randomly between [0, CW].

In [16], authors change the lower bound and upper bound of the backoff interval based on the number of one-hop neighbors and number of transmission attempts. They prove that their algorithm reduces the number of collisions. Authors of [17] enable the nodes to change the upper and lower bounds based on the current network load and past history. In [18], authors introduce different subranges for backoff interval with respect to different network contention levels. Although these methods change their lower and upper bounds, final selection is done randomly within the new bound.

In this paper, we use BEB to tune the CW according to the current contention level. After tuning the CW, we introduce a new method of assigning backoff interval between [0, CW]. The individual parameters of each node like waiting time and queue length are taken into account to compute the backoff interval. These parameters help us to allocate a fair and effective medium access among the nodes. We ensure that unique backoff value is assigned to each node so as to avoid collision. Fuzzy logic is a simple and promising approach that extracts crisp and definite output from vague and ambiguous input parameters. Fuzzy logic has been widely used in wireless communication across various layers for computing, control, and decision making [19]. In [20] authors use fuzzy logic to calculate backoff interval to reduce contention over vehicular ad hoc networks. They control the current backoff interval using the past interval and success ratio of the node. Authors of [21] used fuzzy logic controller for early detection and prevention of congestion at the router buffer. They used delay rate and average queue length as input parameter and produced packet dropping probability as the crisp output. Authors of [22] have active router queue management based on conditions derived from Lyapunov stability theory. They used fuzzy congestion controller for the same.

In our method, each node collects input parameters from contending neighbors. The collected information is processed along with node's own attributes and applied to membership functions to get fuzzy input parameters. By applying these fuzzy variables to the rules base, we can derive the backoff interval as crisp output.

## 3. System Architecture

*Problem with IEEE 802.11*. In IEEE 802.11, the following steps are executed whenever a node wants to transmit a packet.

- Node senses the medium.
- If the medium is idle for distributed interframe space (DIFS) period, then
  - the node generates a backoff counter randomly between [0, CW];
  - the backoff counter is decremented by one for every idle slot;
  - if the channel is busy, the backoff counter is paused until the next DIFS free period;
  - when the backoff counter reaches zero, the node starts transmission.

Each node uses BEB algorithm to find out the current contention window size (CW). The value of CW reflects the contention status of the channel. The minimum and maximum values of CW are called CW_min⁡_ and CW_max⁡_ with the default values of 31 and 1023, respectively. IEEE 802.11 updates CW as follows.

1. CW is initially set to CW_min⁡_.
2. After every unsuccessful transmission CW is doubled with the maximum limit of CW_max⁡_.
3. Upon a successful transmission, CW is reset to CW_min⁡_.

We note that the backoff value is randomly chosen between [0, CW] irrespective of the value of CW. Lower bound 0 changes the optimal order and frequency of channel access among nodes [16–18]. Previous studies have revealed that it greatly affects the average delay and throughput of the individual nodes. For larger number of nodes with heavy traffic, the number of collisions is more which leads to larger value of CW resulting in unfairness [23]. BEB never considers the traffic status (like waiting time or queue length) of the contending nodes for allocating the medium. The number of collisions can be reduced when the contending nodes are assigned with unique backoff value according to its traffic status.

*Proposed Design*. Our proposed algorithm enhances IEEE 802.11 DCF with additional level of contention resolution that prioritizes the contending nodes according to its queue length and waiting time. Each node learns about the contending nodes and computes a unique backoff interval between [0, CW] for itself. Contention window size (CW) is updated using IEEE 802.11 BEB. Each node needs to compute unique backoff interval by comparing its own data with the input parameters collected from contending nodes. In a dense network with larger nodes and heavy traffic, the collected input can be huge and vague. We make use of fuzzy logic at each node to find out its order for accessing medium.

**Figure 1:**
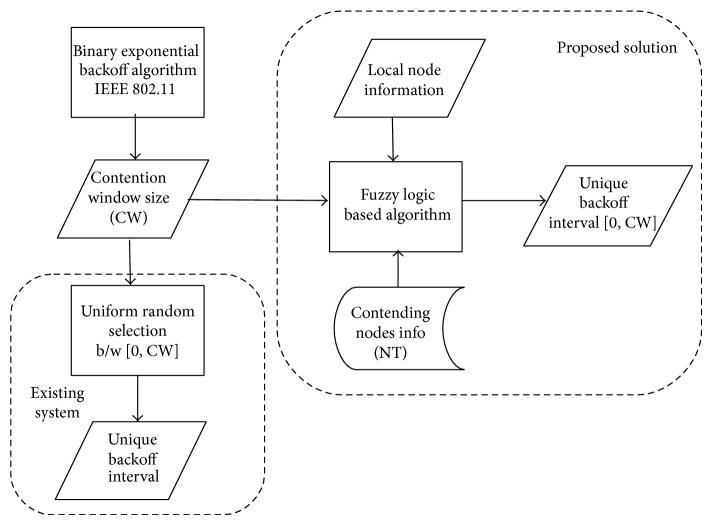
System architecture.

Our system architecture is shown in Figure 1. It is clear that our fuzzy logic algorithm replaces random backoff selection and enhances IEEE 802.11. We specify that our algorithm can be incorporated along with the existing backoff algorithms [4, 6, 7, 9] too. In the next section, we explain our fuzzy logic algorithm in detail.

### 3.1. Fuzzy Logic

**Figure 2:**
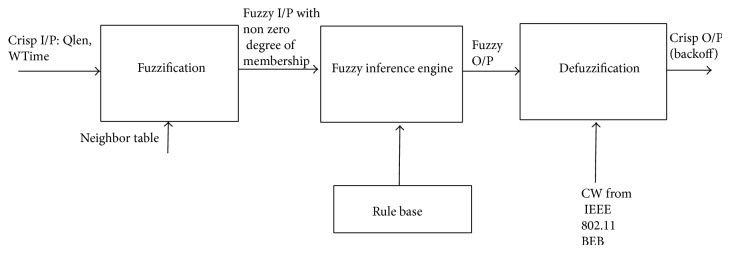
Steps in fuzzy logic.

Fuzzy logic is a promising approach that produces a definite output from vague parameters. The following steps are involved in our algorithm and are shown diagrammatically in Figure 2.

1. Collect the input parameters.
2. Apply the input parameters to the triangular membership functions and retrieve the degree of membership on each fuzzy input variable—fuzzification.
3. Apply the fuzzy values to each rule in the rule base and retrieve the fuzzy output from fuzzy inference engine using Mamdani's method.
4. Defuzzify the fuzzy output to retrieve the crisp output using center of gravity method.

Each step is discussed in detail in the following section.

#### 3.1.1. Collecting Input Parameters

Every node is in the training phase for specific time period after joining the network. When a node wants to transmit a packet during training phase, it executes random BEB for choosing backoff interval between [0, CW]. Each node is responsible for advertising its local data (queue length—myqlen and waiting time—mywt) along with request to send (RTS) message. Nodes that overhear or receive RTS will store the queue length and waiting time of this neighbor in its neighbor table (NT). This NT is dynamically updated every time while receiving or overhearing RTS to reflect the current status of the contending nodes. After the training phase, nodes start using our fuzzy logic algorithm to compute unique backoff interval that is explained in the next section. Updating of NT continues even after training phase. We should ensure that the duration of the training phase is sufficient for each node to overhear information from its neighbors. We have set the training phase based on the number of RTS messages overheard or received. It is set to 15 in our simulation. The steps executed by a node during transmission or reception of RTS packet are shown in Algorithm 1.

In our work, queue length and waiting time have been identified as the input parameters for medium scheduling. Input parameters play an important role in improving the performance of the algorithm. The objective of our algorithm is to have fair and quick ordering among the nodes to access the medium. Competing nodes that are possessing the same contention window size must be given the medium access based on their waiting time. It can definitely improve the fairness and delay. If the node with larger queue length suffers from getting to the media access, it can cause unexpected long delay for the packets waiting in the queue which leads to unnecessary timeout at the respective TCP sources. So, we have taken waiting time and queue length as two input parameters to our fuzzy logic based algorithm. Every node advertises the following information through its RTS message.

*Waiting Time*. Waiting time is defined as the interval between the time at which the current packet entered the queue and time at which it leaves front of the queue.

*Queue Length*. Queue length is defined as the number of packets waiting in the interface queue for transmission. It is measured when the current packet leaves the node and it reflects the total number of packets waiting for medium access.

Whenever a node overhears or receives RTS message from its one-hop neighbors, it updates the collected information in the neighbor table (NT) as shown in Table 1.

### 3.2. Fuzzification

Fuzzification is the process of converting the crisp or scalar input into fuzzy input parameters. Waiting time (mywt) and queue length (myqlen) of the current node are applied as the crisp input to the fuzzification phase. Fuzzy set of our crisp input waiting time takes three values {less, average, more} and fuzzy set of queue length takes the fuzzy values {short, moderate, long}. Fuzzification process applies every crisp input parameter into the membership function of each value of the fuzzy set and finds out degree of membership. It yields us the percentage of membership of the crisp input in each fuzzy value of corresponding fuzzy set.

**Figure 3:**
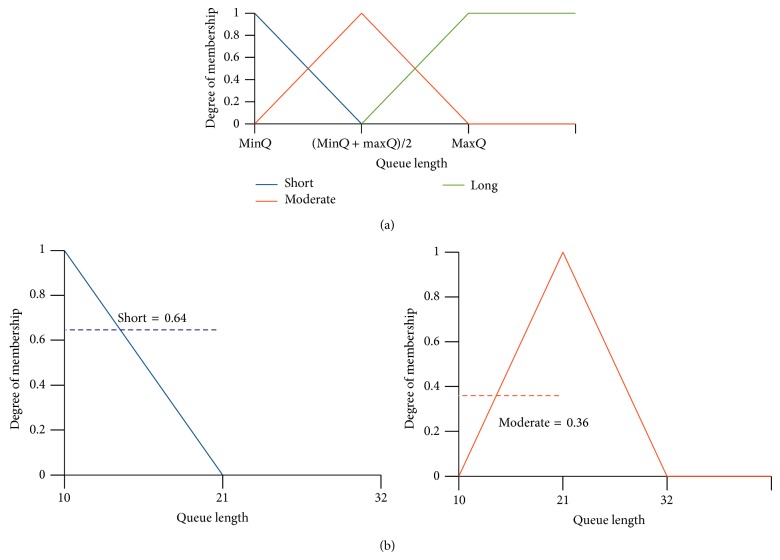
Membership diagram for queue length. (a) and (b) Degree of membership for short and moderate with input myqlen = 14, min⁡*Q* = 10, and max⁡*Q* = 32.

Triangular membership function has been defined for each fuzzy value {short, moderate, long} of queue length in (1). It is used to measure the degree of membership of the given queue length for each fuzzy value. The triangular membership diagram for queue length is shown in Figure 3. In the following equation, myqlen represents the queue length of the local node when the current packet is in front of the queue:

(1) µsmyqlen =1  if  myqlen≤min⁡Qlmin⁡Ql+max⁡Ql/2−myqlenmin⁡Ql+max⁡Ql/2−min⁡Ql   if  min⁡Ql>myqlen<min⁡Ql+max⁡Ql20  if  myqlen≥min⁡Ql+max⁡Ql2  µmmyqlen =myqlen−min⁡Qlmin⁡Ql+max⁡Ql/2−min⁡Ql   if  min⁡Ql≥myqlen<min⁡Ql+max⁡Ql21  if  myqlen=min⁡Ql+max⁡Ql2max⁡Ql−myqlenmax⁡Ql−min⁡Ql+max⁡Ql/2   if  min⁡Ql+max⁡Ql2>myqlen≤max⁡Qlµlmyqlen =0 if  myqlen<min⁡Ql+max⁡Ql2myqlen−min⁡Ql+max⁡Ql/2max⁡Ql−min⁡Ql+max⁡Ql/2  if  min⁡Ql+max⁡Ql2≤myqlen<max⁡Ql1 if  myqlen≥max⁡Ql.

In (1), min⁡*Q*_*l*_ and max⁡*Q*_*l*_ represent the minimum queue length and maximum queue length among contending nodes and they act as the boundary value between fuzzy values. The scalar value of myqlen is compared with min⁡*Q*_*l*_ and max⁡*Q*_*l*_ and a degree of membership between 0 and 1 under each fuzzy value is obtained. *Min*⁡*Q*_*l*_ and max⁡*Q*_*l*_ are calculated from the neighbor table as shown in the following equation and this calculation is done when a node needs backoff counter value just before transmission:

(2) min⁡Ql=minimumQL1,QL2,QL3,…,QLn,max⁡Ql=maximumQL1,QL2,QL3,…,QLn.

In (2), *n* represents the number of neighbors of current node. QL_*i*_ represents the queue length in neighbor *i*. An example for finding out the degree of membership is shown in Figures 3(a) and 3(b). The membership function accepts the queue length of the current node (myqlen) and finds out its degree of membership for each fuzzy value {short, moderate, long} with respect to its contending nodes (min⁡*Q*_*l*_ and max⁡*Q*_*l*_). In this example, myqlen = 14; min⁡*Q*_*l*_ and max⁡*Q*_*l*_ are assumed as 10 and 32, respectively. These values are applied to (1) and the degree of membership for {short, moderate, long} is found to be {0.64,0.36,0}. The membership functions for waiting time can also be derived in the same manner.

### 3.3. Fuzzy Inference Engine

**Figure 4:**
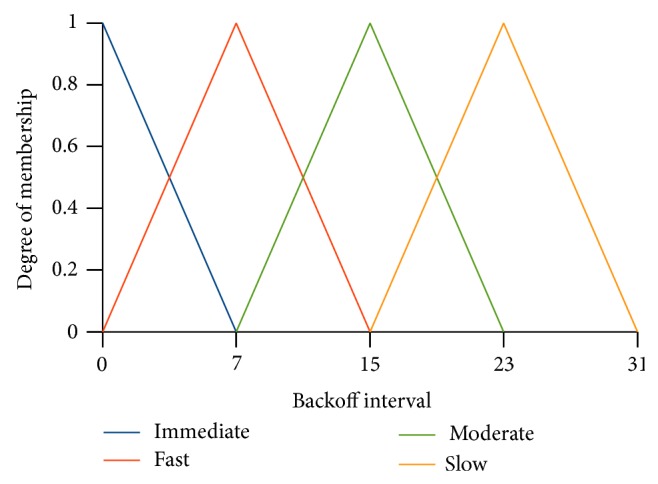
Membership diagram of output parameter backoff interval assuming that CW = 31.

**Figure 5:**
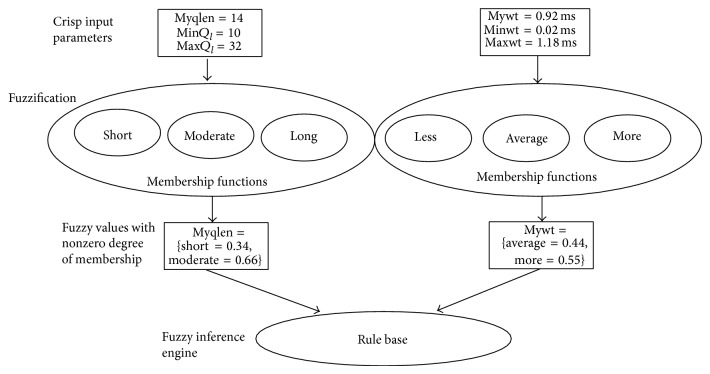
Input to fuzzy inference engine.

Fuzzy inference engine maps the fuzzy input parameters into fuzzy output parameters with the help of rule base. Here, fuzzy input parameter means the fuzzy values that have nonzero degree of membership (e.g., short, moderate from the previous example of Figures 3(a) and 3(b)) from each input parameter. The fuzzy set of our output (medium access) takes four linguistic values {immediate, fast, moderate, slow}. The triangular membership diagram for the fuzzy output is shown in Figure 4. *x*-axis represents the crisp value (backoff) of the fuzzy output. The input parameters to the fuzzy inference engine are shown with an example in Figure 5.

The rule base is the set of rules that reflects the exact behavior of the system. In the rule base, fuzzy value of queue length and waiting time are related to fuzzy value of our output medium access. The rule base is constructed and shown in Table 2. The row represents the possible fuzzy values of waiting time and column represents the fuzzy value of queue length. Each cell represents the fuzzy value of the output parameter medium access which will be mapped to crisp output called backoff interval. The rule is of the form

*if (queue length is long and waiting time is more), then medium access is immediate.*

Since the rule involves two input parameters, we need to evaluate the antecedent of each rule and obtain the membership value for the same. We use conjunction to connect the two parts of the rule. So, we can derive the consequent of each rule by applying the “and” operator to the antecedent.

**Figure 6:**
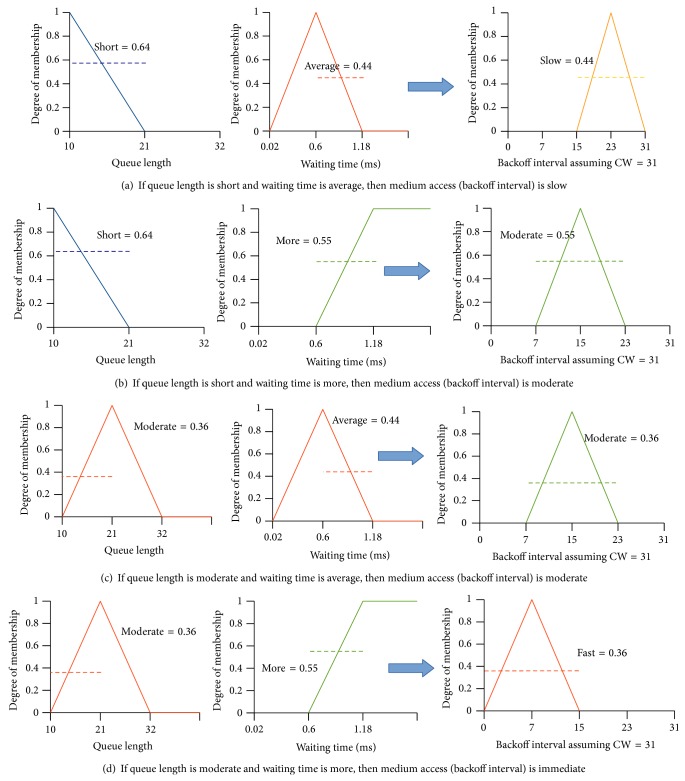
Evaluating the rule base with input parameters myqlen = 14, mywt = 0.92 msec, CW = 31, min⁡*Q*_*l*_ = 10, max⁡*Q*_*l*_ = 32, minWT = 0.02 msec, and maxWT = 1.18 msec.

We recorded the value of input parameters at a specific node during the experiment and we have shown that example diagrammatically for better understanding. It is drawn in Figure 6. We have derived all possible rules of the rule base for the given example. The degree of membership for each fuzzy value of the inputs is applied in the consequent and antecedent is found by applying min operator to the two parts of the consequent.

### 3.4. Defuzzification

**Figure 7:**
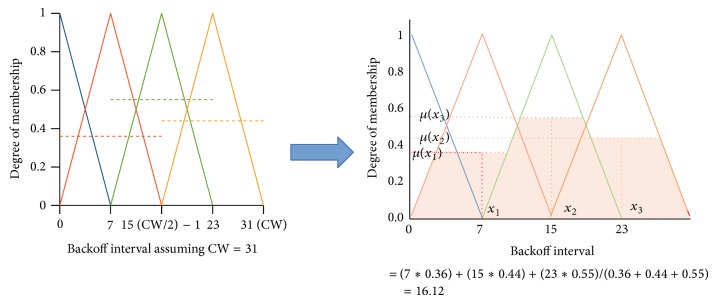
Aggregating the result of Rules with centroid method.

It is the process of mapping the fuzzy output parameters into the crisp output. Our fuzzy output parameter “medium access” takes four linguistic values {immediate, fast, moderate, slow}. The crisp values for these fuzzy set are derived from the CW. Maximum crisp value associated with each fuzzy value of medium access are {0, (CW/4), (CW/2) − 1,3/4∗CW}. The degree of membership for each value has been obtained as the result of evaluating the rules. These membership values are applied to the centroid method of defuzzification to retrieve the crisp output. The formula for the centroid method is shown in (3). Here *x* represents the maximum crisp output value at which the degree of membership is 1 for this fuzzy output and *µ*(*x*) is the membership value of the fuzzy output evaluated from the rule. It is diagrammatically shown in Figure 7:

(3) $$\begin{array}{c} \text{Backoff}=\frac{\int x\cdot {µ}_{\left(r\right)}\left(x\right)dx}{\int {µ}_{\left(r\right)}\left(x\right)}. \end{array}$$

## 4. Performance Evaluation

In this section, we present the simulation results of our proposed system. Our fuzzy approach can be used along with any of existing backoff algorithm to generate a unique backoff interval within CW limit where CW is dynamically updated by backoff algorithm based on channel status. We have incorporated our fuzzy logic algorithm with (i) IEEE 802.11 BEB and named it IEEE802.11 + fuzzy and (ii) GDCF [4] and named it GDCF + fuzzy. Fuzzy approach is implemented as part of MAC 802.11 layer on each node. We have shown the comparison among IEEE802.11, IEEE802.11 + fuzzy, GDCF, and GDCF + fuzzy for various parameters. The simulation is done using ns-2.35 simulator. We have considered two scenarios. In the first scenario 50 nodes are randomly deployed within the area of 500∗500. The second scenario is deployed over 1000∗1000 with 50 nodes. In the third scenario we have tested our protocol by varying both the number of nodes and number of flows over 2000 m∗2000 m area. We have tested our performance for varying loads by changing the number of TCP (transmission control protocol) connections which uses FTP application. The simulation scenario is described in Table 3. We have considered the following parameters for performance evaluation.

### 4.1. Average Throughput

**Figure 8:**
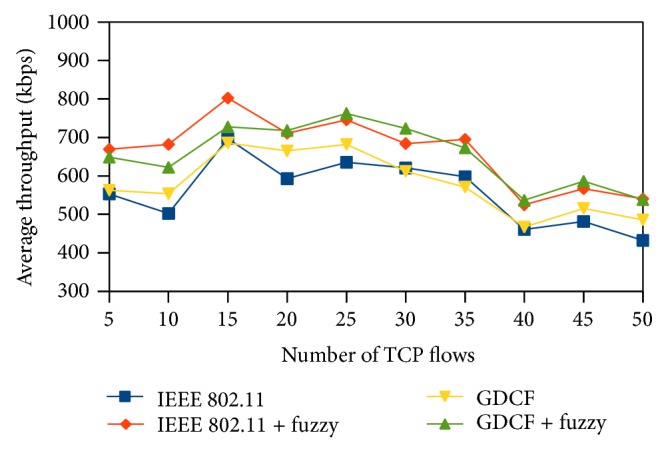
Average throughput for 500∗500 scenario.

**Figure 9:**
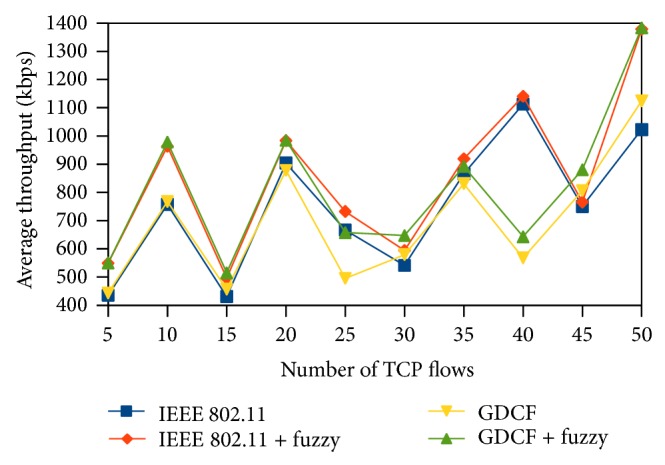
Average throughput for 1000∗1000 scenario.

**Figure 10:**
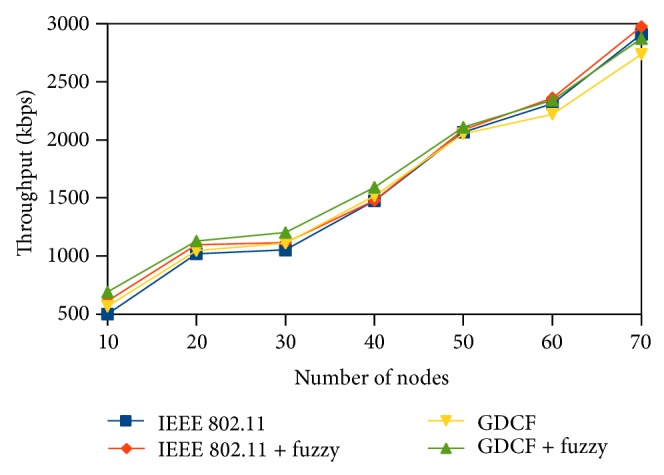
Average throughput for varying nodes.

Throughput is defined as the number of bits received per second by the destination. Average throughput gives us the mean value of throughput for the destination nodes scattered in the network. We measure throughput in terms of kilobits per second. Average throughput of the fuzzy algorithm is better than IEEE 802.11 and GCF. We have plotted the average throughput of scenario 1 and scenario 2 in Figures 8 and 9, respectively. In scenario 1 the nodes are closely placed which results in severe contention and limited spatial reuse. We also observe that throughput does not follow a uniform increase or decrease. In scenario 3, overall throughput is high due to spatial reuse in 2000∗2000. It is shown in Figure 10. Our method performs better than IEEE 802.11 and also GDCF.

### 4.2. Average End to End Delay

**Figure 11:**
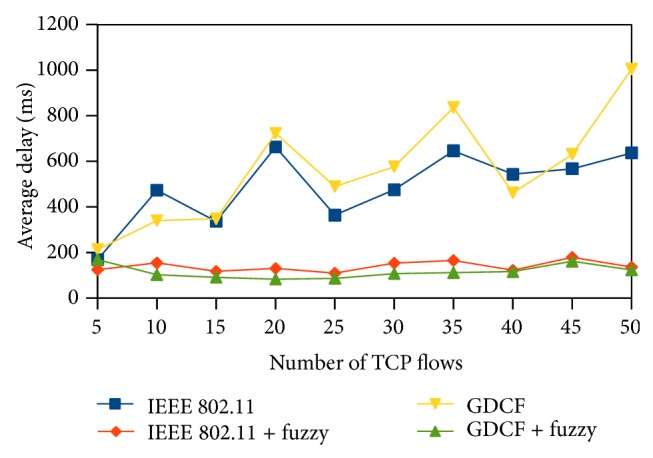
Average end to end delay for 500∗500 scenario.

**Figure 12:**
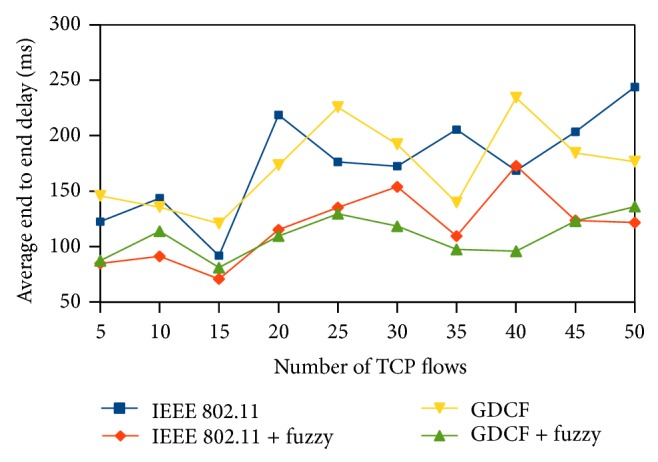
Average end to end delay for 1000∗1000 scenario.

End to end delay is defined as the time period taken for the packet to reach the destination from the source. Average delay gives us the mean delay of the packets transmitted in the end to end path. It is measured in terms of miliseconds. We observe that fuzzy based approaches outperform the others. Contention window size reflects the contention status. CW size will be more when the contention is heavy and less during low traffic. After assigning the CW, nodes are ordered based on their waiting time and queue status. Due to the proper ordering in accessing the medium, every packet is transmitted in a quick manner without much waiting that leads to reduced end to end delay. Approximately our fuzzy methods show 50% reduction in delay in both scenarios as shown in Figures 11 and 12.

### 4.3. Packet Delivery Ratio

**Figure 13:**
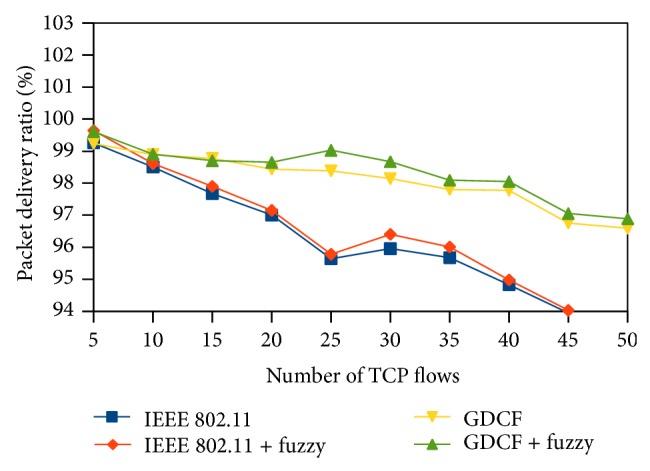
Packet delivery ratio for 500∗500 scenario.

**Figure 14:**
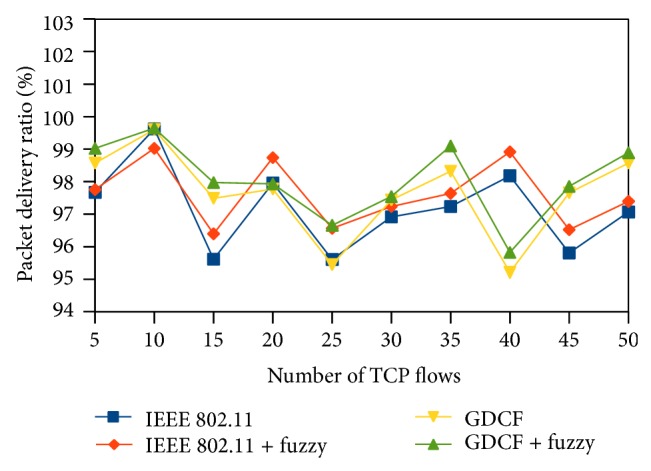
Packet delivery ratio for 1000∗1000 scenario.

**Figure 15:**
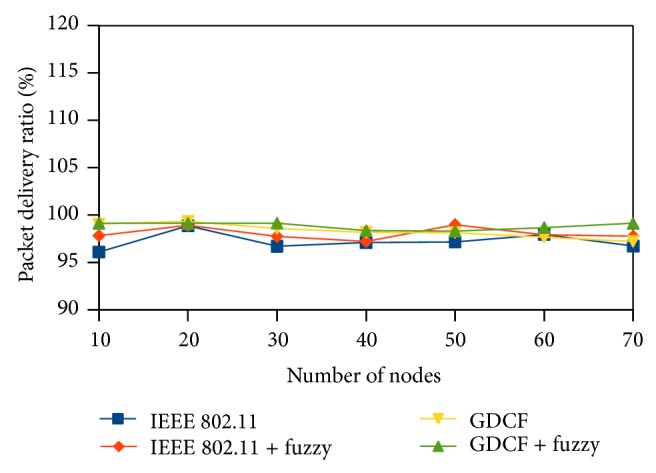
Packet delivery ratio for variable nodes and traffic.

Packet delivery ratio (PDR) represents the ratio of the packets received to the packets transmitted. It is plotted in *y*-axis. Packet delivery ratio reflects the percentage of successful transmission. PDR of fuzzy based approaches is high than IEEE 802.11 and GDCF. It is diagrammatically shown in Figures 13, 14, and 15.

### 4.4. Number of Collisions

Number of collisions is a factor to measure contention in the network. Collisions are more when there is a heavy contention among the nodes to access the medium. It gets increased under increasing loads. These collisions result in unsuccessful transmission that makes the node double the contention window size. Larger contention window size reduces the throughput and increases the delay. The number of collisions in our algorithm is reduced by allocating backoff interval based on nodes buffer status. Nodes with smaller buffer and smaller waiting time make themselves wait, thereby reducing the contention.

The number of collisions also denotes the interferences happened while transmitting two packets at the same time. Collisions also reflect the uniqueness of our fuzzy approach. It is an important factor that determines the uniqueness of backoff interval value generated from our algorithm. It is calculated using the number of collision drops while transmitting request to send (RTS) or clear to send (CTS).

**Figure 16:**
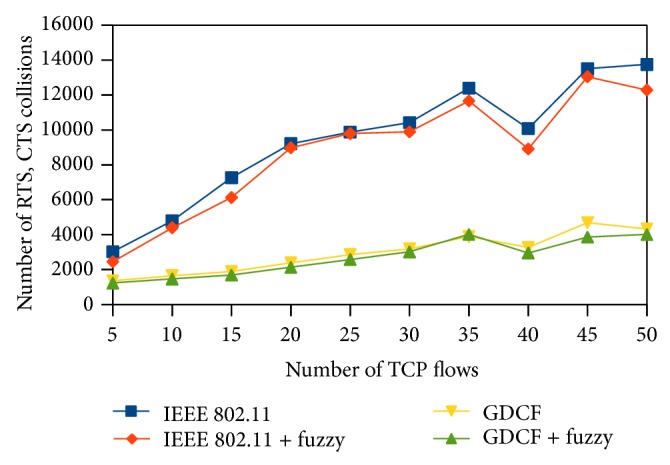
Number of RTS, CTS collisions for 500∗500 scenario.

**Figure 17:**
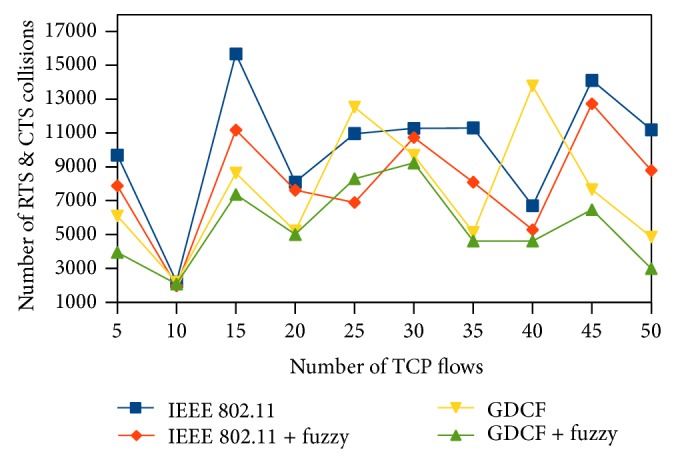
Number of RTS, CTS collisions for 1000∗1000 scenario.

**Figure 18:**
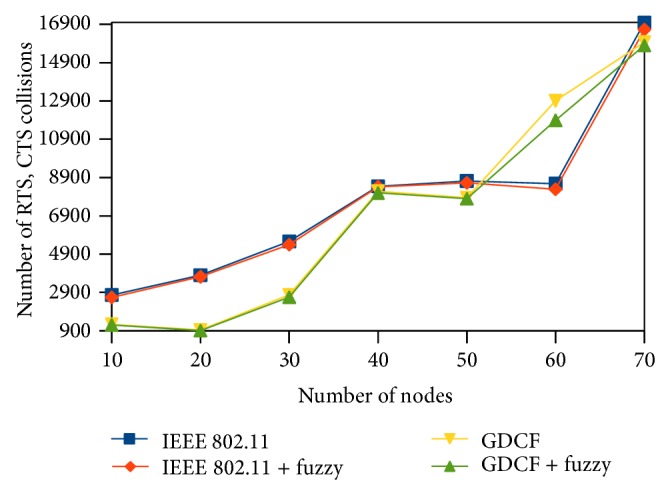
Number of collisions for varying traffic and nodes.

We represent the number of collisions on *y*-axis. Here, we observe that the number of collisions happening in the network is less than random backoff generation algorithms of IEEE 802.11 and GDCF. It is diagrammatically shown in Figures 16, 17, and 18.

## 5. Conclusion

The collision avoidance mechanism of IEEE 802.11 DCF makes it inefficient and unfair especially under heavy load. IEEE 802.11 BEB algorithm makes every node select its backoff interval between [0, CW] in a random and uniform manner. But this random backoff interval can change the optimal order and frequency of channel access among competing nodes which result in unfairness and increased delay. We proposed an algorithm that enables each node to compute its unique backoff interval using fuzzy logic based on the input parameters collected from contending nodes through overhearing. Every node in the network finds its order to access the medium. Our algorithm makes sure that nodes waiting for a long time with more packets get to the medium quickly and nodes with small number of packets and less waiting time get to the medium later. We control the channel contention by ordering the nodes according to their waiting time. Our future work would be to test the performance for dynamic and mobile scenarios and to work on the issues related.

## Figures and Tables

**Algorithm 1:**
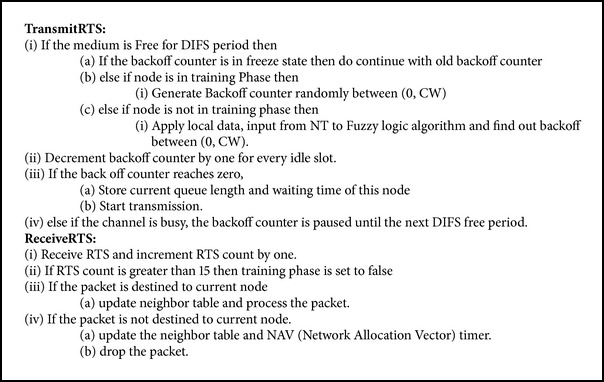
Steps to be followed while transmitting and receiving RTS.

**Table 1:** Neighbor table.

Neighbor ID	Queue length	Waiting time
1	QL_1_	WT_1_
2	QL_2_	WT_2_
3	QL_3_	WT_3_
⋮	⋮	⋮
*n*	QL_*n*_	WT_*n*_

**Table 2:** Rule base for medium access (backoff interval).

			Myqlen	
		Short	Moderate	Long
	Less	*Slow *	*Moderate *	*Fast *
Mywt	Average	*Slow *	*Moderate *	*Immediate *
	More	*Moderate *	*Fast *	*Immediate *

**Table 3:** Simulation setup.

Channel bit Rate	1 Mbps
PLCP data rate	1 Mbps
Backoff slot time	20 *μ*s
CW_min_	31
CW_max_	1023
SIFS	10 *μ*s
DIFS	50 *μ*s
Data packet size	8000 bits
RTS packet size	160 bits + 20 bits additional overhead
Number of nodes	10 to 100
